# Perceived fulfillment of multilevel psychological contract responsibilities, job burnout, and turnover intention among primary care physicians: a structural equation modeling analysis

**DOI:** 10.3389/fpubh.2026.1934893

**Published:** 2026-09-15

**Authors:** Haoyu Zhang, Lifang Zhou, Yifan Jin, Yuexiu Yuan, Leyuan Zhang, Jiahan Li, Xinying Jing, Xingang Sang, Qianqian Yu

**Affiliations:** 1 School of Management, Shandong Second Medical University, Weifang, Shandong, China; 2 School of Public Health, Shandong Second Medical University, Weifang, Shandong, China; 3 Weifang Fangzi District Maternal and Child Health and Family Planning Service Center, Weifang, Shandong, China; 4 Weifang Center for Disease Control and Prevention, Weifang, Shandong, China; 5 Shanghai Institute of Infectious Disease and Biosecurity, Fudan University, Shanghai, China

**Keywords:** family doctors, JD-R model, job burnout, occupational mental health, psychological contract fulfillment, turnover intention

## Abstract

**Background and objectives:**

Primary care physicians face substantial occupational mental health challenges, including job burnout and turnover intention. Psychological contract theory conceptualizes employment relationships in terms of perceived reciprocal obligations, while the Job Demands–Resources (JD-R) model provides a complementary framework for understanding how work-related conditions may be associated with burnout. This study examined the structural associations among perceived fulfillment of multilevel psychological contract responsibilities, job burnout dimensions, and turnover intention among primary care physicians.

**Methods:**

A cross-sectional survey was conducted among 486 family doctors from primary healthcare institutions in Shandong Province using multi-stage random sampling. Perceived psychological contract fulfillment was assessed across three responsibility domains: institutional responsibility, physicians’ responsibility, and governmental/societal responsibility. Structural equation modeling (SEM) and bootstrap analysis with 5,000 resamples were used to examine direct and indirect associations among responsibility fulfillment, burnout dimensions, and turnover intention.

**Results:**

Perceived fulfillment of the three psychological contract responsibility domains showed differentiated associations with burnout dimensions and turnover intention. Institutional responsibility demonstrated a significant negative direct association with turnover intention (*β* = −0.231, *p* < 0.01) as well as significant indirect associations through emotional exhaustion and cynicism. Governmental/societal responsibility was indirectly associated with lower turnover intention primarily through cynicism (*β* = −0.141, *p* < 0.01) and secondarily through emotional exhaustion (*β* = −0.036, *p* < 0.05). Physicians’ responsibility showed no significant total association with turnover intention but demonstrated a significant specific indirect association through reduced personal accomplishment (*β* = −0.025, *p* < 0.01).

**Conclusion:**

Perceived fulfillment of different psychological contract responsibility domains was differentially associated with burnout dimensions and turnover intention among primary care physicians. Institutional and governmental/societal responsibility fulfillment showed associations primarily involving emotional exhaustion and cynicism, whereas physicians’ responsibility was specifically associated with the pathway involving reduced personal accomplishment. These cross-sectional findings should be interpreted as statistical associations, and longitudinal studies are needed to clarify their temporal and causal relationships.

## Introduction

The retention of primary care physicians (PCPs) has emerged as a critical global public health challenge, as workforce shortages and severe occupational stress threaten the sustainability of healthcare infrastructure worldwide. Within this global context, strengthening primary care is a cornerstone of China’s ongoing healthcare reforms, with the family doctor contract service system serving as the primary mechanism for establishing a hierarchical medical system (1). As the frontline “gatekeepers,” family doctors are tasked with an expansive range of responsibilities, including chronic disease management, preventive care, and clinical treatment (2). However, the rapid expansion of these services has not been matched by a proportional increase in systematic support, leaving many PCPs in a state of chronic professional stress (3). High-intensity workloads, stagnant compensation, and the persistent pressure of patient expectations have created a significant occupational mental health burden among this workforce (4, 5). Within this context, the distribution of responsibilities across healthcare institutions, physicians themselves, and governmental/societal actors provides a relevant framework for examining the occupational experiences of primary care physicians (6).

Job burnout—a psychological syndrome characterized by emotional exhaustion, cynicism (depersonalization), and a sense of reduced personal accomplishment—has emerged as a critical threat to the mental well-being of healthcare professionals (7, 8). Beyond its clinical implications for the individual physician, such as impaired well-being and psychological distress, burnout is associated with organizational instability and higher turnover intention (9, 10). In the relationship-intensive context of primary care, burnout has also been associated with poorer physician–patient interactions and challenges to the continuity and quality of care (11–13). Understanding the factors associated with burnout and its relationship with subsequent exit cognitions is therefore important for addressing primary care workforce sustainability.

Psychological contract theory provides the primary conceptual foundation for the responsibility domains examined in this study. Psychological contracts refer to individuals’ beliefs regarding reciprocal obligations within employment relationships (14). In the Chinese healthcare context, previous research has extended this framework beyond the immediate physician–institution relationship and conceptualized physicians’ psychological contracts across three responsibility domains: institutional responsibility, physicians’ own responsibility, and governmental/societal responsibility (6). Importantly, the present study focuses on the perceived fulfillment of these responsibilities. Respondents evaluated the extent to which responsibilities within each domain had been fulfilled; therefore, the three domains represent perceived fulfillment of multilevel psychological contract responsibilities rather than different sources of psychosocial support. Previous research has linked psychological contract fulfillment and related contract perceptions with emotional experiences, burnout-related outcomes, and turnover intention (15–17).

The JD-R model is used in the present study as a complementary explanatory framework rather than as a basis for redefining psychological contract fulfillment as a job resource. The JD-R model proposes that occupational well-being reflects the balance between job demands and available job and personal resources (18–20). From this perspective, fulfillment of institutional and governmental/societal responsibilities may be relevant to burnout because these domains involve work-related conditions such as employment security, compensation, working conditions, professional protection, policy support, and social recognition. However, these responsibility domains are not operationally equivalent to job resources; rather, their fulfillment may indicate favorable contextual conditions that have resource-related implications for physicians’ work experiences. Physicians’ own responsibility requires a different interpretation. Because this domain concerns physicians’ perceptions of the extent to which they themselves fulfill professional obligations, it should not be regarded as external organizational or system support. Nor do we operationalize physicians’ responsibility itself as a personal resource. Instead, fulfillment of physicians’ professional obligations may be associated with experiences related to competence, professional efficacy, role consistency, or professional values, which may in turn be relevant to burnout-related experiences. These potential resource-related implications are therefore treated as theoretical interpretations rather than as part of the operational definition of physicians’ responsibility. Psychological contract fulfillment should also be distinguished from perceived organizational support (POS). POS refers to employees’ general beliefs that their organization values their contributions and cares about their well-being (21). In contrast, psychological contract fulfillment concerns the perceived extent to which reciprocal obligations are fulfilled. In the present study, these obligations are assessed across institutional, physicians’, and governmental/societal responsibility domains. POS was not directly measured and is therefore treated as a conceptually related but distinct construct rather than as an alternative label for the variables examined in this study.

Consistent with the JD-R framework, previous research has shown that job demands and work-related resources are differentially associated with burnout and withdrawal-related outcomes (19, 22, 23). While prior research has explored factors such as workload and income in relation to family doctors’ turnover intention (24, 25), comparatively less attention has been paid to how perceived fulfillment of different psychological contract responsibility domains is associated with burnout and turnover intention.

Turnover intention, a conscious plan to leave an organization, is a key precursor of actual turnover behavior (26). It has been closely linked to emotional exhaustion and perceived imbalances between effort and reward (27–29). However, the differentiated indirect associations linking fulfillment of specific psychological contract responsibility domains, distinct burnout dimensions, and turnover intention remain insufficiently understood in primary healthcare settings (30, 31).

Despite extensive research on turnover intention in primary healthcare, most studies have focused on individual predictors such as workload, income, or job satisfaction. Less attention has been given to perceived fulfillment of psychological contract responsibilities across multiple responsibility domains and to whether these domains show differentiated associations with the three dimensions of burnout. In particular, the indirect associations involving emotional exhaustion, cynicism, and reduced personal accomplishment across institutional, physicians’, and governmental/societal responsibility domains remain insufficiently understood. To address these gaps, this study integrates psychological contract theory with the JD-R model to examine the structural associations among perceived fulfillment of multilevel psychological contract responsibilities, burnout dimensions, and turnover intention among family doctors. Specifically, we aimed to: (1) examine the associations between fulfillment of institutional, physicians’, and governmental/societal responsibilities and burnout dimensions; (2) examine differentiated indirect associations through emotional exhaustion, cynicism, and reduced personal accomplishment; and (3) characterize the relationships between these responsibility domains and turnover intention.

## Literature review and hypothesis construction

Psychological contract research suggests that perceptions of fulfilled or unfulfilled obligations are associated with employees’ emotional and work-related outcomes, including burnout-related experiences and turnover intention (15–17, 32–36). Within the JD-R framework, job demands and work-related resources are likewise associated with burnout and withdrawal-related outcomes (19, 20, 23, 37). In the present study, the JD-R model is therefore used to provide a complementary explanation for why perceived fulfillment of psychological contract responsibilities may be relevant to burnout, rather than to redefine psychological contract fulfillment itself as a job resource. Burnout may, in turn, represent a statistical indirect pathway in the association between perceived responsibility fulfillment and turnover intention (10, 15, 37) (Figure 1). Importantly, burnout is a multidimensional construct involving emotional exhaustion, cynicism, and reduced personal accomplishment, which represent conceptually distinct responses to occupational stress (8, 38). Emotional exhaustion reflects the depletion of emotional and energetic resources, cynicism reflects psychological detachment and negative attitudes toward work, whereas reduced personal accomplishment reflects diminished feelings of competence and professional efficacy. Previous research has further suggested that psychosocial work factors may show differential associations with these three burnout dimensions (39).

**Figure 1:**
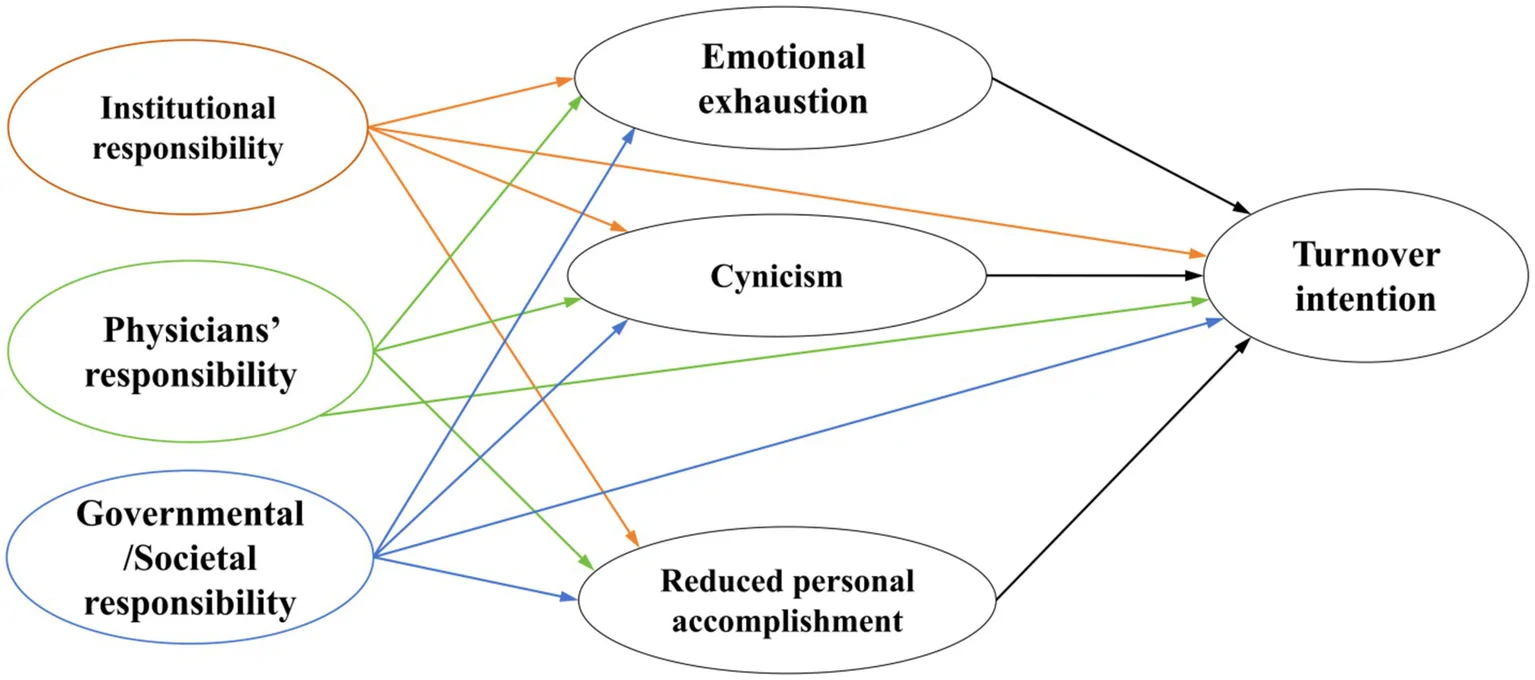
Hypothetical conceptual framework.

Institutional responsibility concerns obligations located primarily within physicians’ immediate employing environment, including employment security, compensation, working conditions, professional protection, work organization, development opportunities, and organizational cooperation (6). Because these obligations concern proximal features of physicians’ everyday work context, their perceived fulfillment may be particularly relevant to emotional exhaustion and cynicism. Emotional exhaustion reflects depletion under sustained occupational demands, whereas cynicism involves psychological distancing from one’s work and work environment. Consistent with this interpretation, previous physician research has shown that organizational and psychosocial work factors may exhibit particularly relevant associations with exhaustion and cynicism (39, 40).

Governmental/societal responsibility concerns obligations located in the broader professional environment, including policy and financial support, occupational protection, social recognition, patient trust, and fair treatment of physicians within the wider healthcare system (6). Compared with institution-level obligations, fulfillment of these broader responsibilities may be more closely related to physicians’ evaluations of the legitimacy, recognition, and sustainability of their professional environment. Such evaluations may be particularly relevant to cynicism, which involves psychological detachment and negative attitudes toward work, and may also be associated with emotional exhaustion when unfavorable broader conditions coincide with high occupational demands. Research on physician burnout has similarly indicated that work-related demands, values, and broader psychosocial conditions may show differentiated associations with burnout dimensions (39, 41).

Physicians’ responsibility differs conceptually from the other two domains because it concerns physicians’ perceptions of the extent to which they themselves fulfill professional obligations, including adherence to professional standards, completion of assigned duties, continued professional development, cooperation with colleagues, and commitment to professional and organizational responsibilities (6). This domain therefore should not be interpreted as external support, nor is physicians’ responsibility itself operationalized as a personal resource in the present study. However, because fulfillment of these obligations concerns physicians’ own professional functioning and standards, it may be more closely related to experiences of competence, professional efficacy, value congruence, and accomplishment. These experiences are conceptually relevant to reduced personal accomplishment, which reflects diminished perceptions of professional efficacy and achievement. Previous physician research has suggested that value-related and individual characteristics may show particularly relevant associations with professional efficacy-related burnout outcomes (41, 42).

Taken together, these conceptual distinctions suggest that perceived fulfillment of the three responsibility domains may not be uniformly associated with all dimensions of burnout. Institutional and governmental/societal responsibilities concern conditions located primarily in the immediate organizational and broader professional environments, respectively, whereas physicians’ responsibility concerns the physician’s own fulfillment of professional obligations. Given the conceptual distinctions among emotional exhaustion, cynicism, and reduced personal accomplishment, these responsibility domains may therefore show differentiated patterns of association with burnout dimensions. This provides a theoretical basis for examining whether the three burnout dimensions exhibit different statistical indirect associations between perceived fulfillment of the responsibility domains and turnover intention. These proposed differences are treated as theory-informed expectations rather than established causal mechanisms.

Unlike previous studies that have focused primarily on individual organizational or occupational factors, the present study examines perceived fulfillment of psychological contract responsibilities across institutional, physicians’, and governmental/societal domains and their differentiated associations with burnout dimensions and turnover intention among family doctors. Based on these theoretical considerations, we propose the following hypotheses:

*Hypothesis 1* Higher perceived fulfillment of institutional, physicians’, and governmental/societal responsibilities is associated with lower levels of job burnout among family doctors.

*Hypothesis 2* Higher levels of job burnout are associated with greater turnover intention among family doctors.

*Hypothesis 3* Higher perceived fulfillment of institutional, physicians’, and governmental/societal responsibilities is associated with lower turnover intention among family doctors.

*Hypothesis 4* Emotional exhaustion, cynicism, and reduced personal accomplishment exhibit differentiated indirect associations between perceived fulfillment of the three psychological contract responsibility domains and turnover intention.

### Methods

#### Ethics approval and consent to participate

This study was approved by the Ethics Committee of Shandong Second Medical University (SDSMU) (Approval No. 2025TX177) and adhered to the Declaration of Helsinki. All participants provided written informed consent prior to participation.

## Research environment and subjects

This cross-sectional study was conducted in Shandong Province, eastern China. In 2025, Shandong ranked among the leading provinces in China in terms of gross domestic product. The province has also established a relatively well-developed network of community health service centers and has made substantial progress as a national demonstration province for integrated medical and older adult care services (43). These characteristics provide a relevant socioeconomic and primary healthcare context for the present study.

A multistage random sampling method was employed to enhance the geographical and socioeconomic diversity of the sample. At the first stage, cities were stratified according to their level of economic development, and three cities representing relatively high, medium, and low levels of economic development were randomly selected. At the subsequent stage, primary healthcare institutions within the three selected cities were further stratified according to the economic development level of their respective areas. Institutions representing relatively high, medium, and low levels of economic development were then randomly selected from these strata, resulting in a total of 39 primary healthcare institutions. This stratified multistage sampling strategy was designed to capture variation in socioeconomic conditions and primary healthcare settings within Shandong Province. Finally, 514 family doctors from these institutions were invited to participate. To ensure data quality, questionnaires were distributed on-site by trained investigators, and participants were informed of the study’s anonymity and academic purpose. Using the Kendall empirical estimation method (5–10 times the number of measurement items), a minimum sample size of 455 was required. A total of 486 valid questionnaires were returned (effective response rate: 94.55%), meeting the requirements for robust structural equation modeling.

### Measurement tools

To ensure contextual appropriateness and item clarity in the primary care setting, the measurement instruments were reviewed by a panel of five experts in health management and psychology, followed by a pilot study among 20 family doctors. The review and pilot testing were used to refine contextual wording and item comprehensibility rather than to establish a new measurement construct.

The psychological contract was assessed using the instrument developed by Shi et al. (6) for physicians working in Chinese public hospitals. The original instrument comprises three responsibility domains: institutional responsibility (24 items), physicians’ responsibility (14 items), and governmental/societal responsibility (13 items). In the present study, the instrument was applied to family doctors working in primary healthcare institutions. Wording referring specifically to hospital-based organizational contexts was therefore contextually adapted to primary healthcare settings, while the original tripartite responsibility structure was retained. No items were deleted during the adaptation process or before data collection. Because the original instrument was developed in Chinese and was administered in the same linguistic context, no translation or back-translation procedure was required.

Respondents rated each item on a six-point scale according to the perceived degree to which the corresponding responsibility had been fulfilled, ranging from 1 (“not fulfilled”) to 6 (“fully fulfilled”), with higher scores indicating a higher perceived degree of fulfillment. Thus, although the three subscales retain their original labels of institutional responsibility, physicians’ responsibility, and governmental/societal responsibility, the scores analyzed in the present study represent perceived fulfillment of these psychological contract responsibility domains. In the present research sample, the overall Cronbach’s α coefficient of the psychological contract measurement instrument reached 0.972.

Job Burnout: Burnout was assessed using the Chinese version of the Maslach Burnout Inventory validated by Li and Shi (44), which has demonstrated adequate psychometric properties in Chinese healthcare settings (45, 46). The scale measures three dimensions: emotional exhaustion, cynicism, and reduced personal accomplishment. Minor contextual modifications were made to ensure the items reflected the specific working conditions of family physicians. The overall Cronbach’s alpha was 0.889.

Turnover Intention: Turnover intention was measured using the four-item scale developed by Farh et al. (47), focusing on quit cognitions and future work plans. This scale has been widely utilized in research among Chinese public servants (48). The Cronbach’s alpha in this study was 0.715.

### Statistical analysis

Data were analyzed using SPSS 25.0 and AMOS 28.0. Descriptive statistics and Pearson correlation analysis were conducted to examine the relationships among variables. We then constructed a structural equation model (SEM). No item parceling procedure was applied in the SEM analysis. All latent constructs were modeled using their retained measurement items as observed indicators after item screening and model refinement. To ensure the stability of the latent constructs, we followed a pre-specified psychometric protocol where items with standardized factor loadings below 0.60 were considered for deletion to optimize the measurement model. Model fit was evaluated using *χ^2^/df*, CFI, TLI, RMSEA, and SRMR. Demographic and occupational characteristics, including age, working hours, employment status, salary, incentive policies, and health status, were collected and summarized descriptively. These variables were not included in the primary structural model because the prespecified analytical focus was on the latent associations among psychological contract responsibility fulfillment, burnout dimensions, and turnover intention. Accordingly, the estimated structural associations should be interpreted as unadjusted for these demographic and occupational characteristics, and the possibility of residual confounding is acknowledged as a study limitation. Finally, bootstrap analysis with 5,000 resamples was used to estimate indirect associations and their 95% confidence intervals among psychological contract responsibility fulfillment, burnout dimensions, and turnover intention. Given the cross-sectional design, these indirect associations were interpreted as statistical associations rather than evidence of temporal or causal mediation. Given that all variables were collected through self-reported questionnaires at a single time point, potential common method bias (CMB) was assessed (49). Harman’s single-factor test was conducted by entering all measurement items into an exploratory factor analysis, and the proportion of variance explained by the first factor was examined.

## Results

### Descriptive analysis

Table 1 details the sociodemographic and work characteristics of the 486 participating family doctors. The sample was predominantly female (70.0%) and aged 35 or older (81.3%). Notably, a significant proportion of the respondents faced challenging work conditions: the majority reported working 8–10 h daily (56.1%), earning 5,000 RMB or less per month (55.8%), and lacking institutional incentive measures (56.6%). Furthermore, 59.3% were employed under contract status (extramural personnel relationships) (Table 1).

**Table 1:** Sociodemographic characteristics of study participants (*N* = 486).

Variables	Frequency (n)	Percent (%)
Gender		
Male	146	30
Female	340	70
Age		
<30	37	7.6
30–34	54	11.1
35–39	99	20.4
40–44	101	20.8
45–49	89	18.3
≥ 50	106	21.8
Marital Status		
Unmarried and Other	55	11.3
Married	431	88.7
Educational Level		
Graduate Degree	18	3.7
Bachelor’s Degree	339	69.8
Below Bachelor’s Degree	129	26.6
Professional Title		
Undetermined Title	39	8
Junior	150	30.9
Intermediate	219	45.1
Associate Senior	72	14.8
Senior	6	1.2
Monthly Income (CNY)		
≤5,000	271	55.8
5,000–10,000	210	43.2
10,000–15,000	5	1
Years of Service in the Institution		
≤5	143	29.4
6–10	122	25.1
11–15	68	14
>15	153	31.5
Average Daily Working Hours (hours)		
<8	195	40.1
8–10	273	56.1
≥10	18	3.8
Incentive Measures Provided by the Institution		
Yes	211	43.4
No	275	56.6
Employment Status		
Regular Staff	198	40.7
Contract Staff	288	59.3
Health Status		
Poor	7	1.4
Fair	113	23.3
Good	366	75.3

### Reliability and validity analysis

Before measurement-model refinement, the overall Cronbach’s α coefficients were 0.972 for the psychological contract instrument, 0.889 for the burnout instrument, and 0.715 for the original turnover intention scale (50). Composite reliability (CR) values for the latent constructs ranged from 0.925 to 0.963 (51). Convergent validity was assessed based on standardized factor loadings and average variance extracted (AVE). In the initial measurement model before item refinement, standardized factor loadings ranged from 0.50 to 0.92. Furthermore, the AVE values for all constructs ranged from 0.514 to 0.767, entirely surpassing the recommended threshold of 0.50. Given these robust parameter ranges, the overall convergent validity for these contextual constructs is considered adequate (52, 53). Discriminant validity was assessed using the Fornell–Larcker criterion. As shown in Table 2, the square root of AVE for each construct exceeded the corresponding correlations between constructs (51), indicating satisfactory discriminant validity among the latent variables.

**Table 2:** Discriminant validity assessment using the Fornell–Larcker criterion.

Construct	IR	PR	GR	EE	CYN	RPA	TI
Institutional responsibility	0.734						
Physicians’ responsibility	0.389	0.772					
Governmental/ societal responsibility	0.675	0.240	0.817				
Emotional exhaustion	−0.417	−0.133	−0.394	0.876			
Cynicism	−0.473	−0.243	−0.516	0.648	0.869		
Reduced personal accomplishment	−0.193	−0.234	−0.189	0.154	0.300	0.865	
Turnover intention	−0.484	−0.156	−0.439	0.529	0.627	0.291	0.847

IR, institutional responsibility; PR, physicians’ responsibility; GR, governmental/societal responsibility; EE, emotional exhaustion; CYN, cynicism; RPA, reduced personal accomplishment; TI, turnover intention.

### Common method bias assessment

Given that all variables were collected from the same respondents using self-reported questionnaires, Harman’s single-factor test was conducted to assess potential common method bias. The results showed that the first unrotated factor accounted for 35.025% of the total variance, which was below the predefined 40% threshold. This result suggests that no single factor accounted for the majority of covariance among the measures and that common method bias was unlikely to constitute a major concern in the present study. Nevertheless, the possibility of residual common method bias cannot be completely excluded.

### Correlation analysis

As shown in Table 3, perceived fulfillment of institutional, physicians’, and governmental/societal responsibilities was significantly positively correlated across the three psychological contract responsibility domains. Higher perceived fulfillment of the responsibility domains was generally associated with lower levels of job burnout, providing preliminary support for Hypothesis 1. Job burnout dimensions were positively associated with turnover intention, with cynicism showing the strongest correlation, supporting Hypothesis 2. Higher perceived fulfillment of institutional and governmental/societal responsibilities showed comparatively stronger negative correlations with turnover intention, whereas the correlation involving physicians’ responsibility was weaker. Overall, the correlation results provided preliminary evidence for the proposed associations among psychological contract responsibility fulfillment, burnout dimensions, and turnover intention (Table 3).

**Table 3:** Correlations among psychological contract responsibility fulfillment, job burnout, and turnover intention.

Variables	*M* ± *SD*	(1)	(2)	(3)	(4)	(5)	(6)	(7)
(1) Institutional responsibility	5.07 ± 0.86							
(2) Physicians’ responsibility	5.69 ± 0.47	0.424^**^						
(3) Governmental/Societal responsibility	4.89 ± 0.93	0.646^**^	0.288^**^					
(4) Emotional exhaustion	2.87 ± 1.05	−0.400^**^	−0.156^**^	−0.369^**^				
(5) Cynicism	2.10 ± 1.02	−0.445^**^	−0.262^**^	−0.472^**^	0.597^**^			
(6) Reduced personal accomplishment	2.84 ± 1.39	−0.152^**^	−0.212^**^	−0.142^**^	0.124^**^	0.238^**^		
(7) Turnover intention	1.87 ± 0.77	−0.426^**^	−0.203^**^	−0.356^**^	0.431^**^	0.552^**^	0.265^**^	

^*^p < 0.05, ^**^p < 0.01.

## Structural associations among psychological contract responsibility fulfillment, burnout, and turnover intention

### Explanation of the revised model

Before examining the hypothesized indirect associations using structural equation modeling, the measurement model was evaluated based on the JD-R theoretical framework (54). During measurement model refinement, items with standardized factor loadings below 0.60 were considered for removal based on established recommendations (55, 56). Specifically, two items from the physicians’ responsibility dimension (F6 and F8) and one item from the turnover intention scale (I3) were removed because their standardized factor loadings did not meet the recommended threshold, suggesting weaker representation of the corresponding latent constructs. In the final measurement model, standardized factor loadings for all retained items ranged from 0.60–0.92. No items were removed from the institutional responsibility, governmental/societal responsibility, or burnout dimensions. Following item removal, internal consistency was reassessed using the retained items. The overall Cronbach’s α coefficient of the psychological contract instrument was 0.971 after removal of F6 and F8. The Cronbach’s α coefficient of the final three-item turnover intention scale was 0.795 after removal of I3. No burnout items were removed, and its overall Cronbach’s α coefficient remained 0.889. Item removal was conducted as a measurement refinement procedure rather than solely to improve model fit. After item removal, the revised measurement model was re-evaluated by examining construct reliability, convergent validity, discriminant validity, and overall model fit. Modification indices were examined during model refinement. Residual covariances were specified between F2-F3 (physicians’ responsibility) and G1-G2 (governmental/societal responsibility) based on statistical and conceptual considerations. No additional structural paths or parameter constraints were introduced, and the hypothesized model structure remained unchanged. The structural equation model demonstrated acceptable overall fit (*χ^2^/df* = 2.384, CFI = 0.900, TLI = 0.896, RMSEA = 0.053, SRMR = 0.048). In Table 4, *χ^2^/df*, CFI, RMSEA, and SRMR met commonly accepted criteria. Although the TLI value was slightly below 0.90, it remained close to the recommended threshold, suggesting an acceptable overall model fit.

**Table 4:** Model fit indices of the structural equation model.

Fit index	Recommended criterion	Observed value
*χ^2^*/df	<3.00	2.384
CFI	>0.90	0.900
TLI	>0.90	0.896
RMSEA	<0.08	0.053
SRMR	<0.08	0.048

### Bootstrap test

The structural pathways were tested by combining structural equation modeling (SEM) with 5,000 Bootstrap resamples. Perceived fulfillment of institutional, physicians’, and governmental/societal responsibilities was entered as the three predictor domains, emotional exhaustion, cynicism, and reduced personal accomplishment as parallel intermediate variables, and turnover intention as the outcome variable. Direct, total, and specific indirect associations were estimated with 95% confidence intervals.

Perceived fulfillment of institutional responsibility showed a significant negative total association with turnover intention (*β* = −0.360, *p* < 0.01, 95% CI: −0.492 to −0.224). The direct association was significant (*β* = −0.231, *p* < 0.01, 95% CI: −0.351 to −0.110), and the total indirect association was also significant (*β* = −0.129, *p* = 0.003, 95% CI: −0.218 to −0.046). Significant specific indirect associations were observed through emotional exhaustion (*β* = −0.050, *p* = 0.033, 95% CI: −0.122 to −0.003) and cynicism (*β* = −0.074, *p* = 0.009, 95% CI: −0.142 to −0.017) whereas the indirect association through reduced personal accomplishment was not significant (*β* = −0.005, *p* = 0.553, 95% CI: −0.026 to 0.015).

Perceived fulfillment of physicians’ responsibility showed no significant total association with turnover intention (*β* = 0.032, *p* = 0.544, 95% CI: −0.081 to 0.119). Neither the direct effect (*β* = 0.084, *p* = 0.087, 95% CI: −0.015 to 0.178) nor the total indirect association (*β* = −0.052, *p* = 0.081, 95% CI: −0.127 to 0.007) was significant. However, the specific indirect association through reduced personal accomplishment was significant (*β* = −0.025, *p* = 0.002, 95% CI: −0.054 to −0.007), whereas the indirect associations through emotional exhaustion (*β* = 0.005, *p* = 0.707, 95% CI: −0.019 to 0.031) and cynicism (*β* = −0.031, *p* = 0.057, 95% CI: −0.084 to 0.001) were not significant.

Perceived fulfillment of governmental/societal responsibility showed a significant negative total association with turnover intention. The direct effect was not significant, whereas the total indirect effect was significant, indicating that the structural relationship was primarily driven by indirect pathways. Highly significant indirect association were found through cynicism (*β* = −0.141, *p* < 0.01, 95% CI: −0.228 to −0.071) and emotional exhaustion (*β* = −0.036, *p* = 0.039, 95% CI: −0.076 to −0.002), whereas the indirect association through reduced personal accomplishment was not significant (*β* = −0.016, *p* = 0.067, 95% CI: −0.043 to 0.001). The three burnout dimensions showed differentiated specific indirect associations across the psychological contract responsibility domains, providing support for Hypothesis 4. The detailed data are displayed in Table 5.

**Table 5:** Decomposition table of total effect, direct effect, and indirect effect.

Path	Standardized effect (β)	*P*	SE	95%CI (lower)	95%CI (upper)
X1-M1-Y	−0.050	0.033	0.031	−0.122	−0.003
X1-M2-Y	−0.074	0.009	0.032	−0.142	−0.017
X1-M3-Y	−0.005	0.553	0.010	−0.026	0.015
Direct Effect	−0.231	<0.01	0.062	−0.351	−0.110
Indirect Effect	−0.129	0.003	0.044	−0.218	−0.046
Total Effect	−0.360	<0.01	0.068	−0.492	−0.224
X2-M1-Y	0.005	0.707	0.012	−0.019	0.031
X2-M2-Y	−0.031	0.057	0.022	−0.084	0.001
X2-M3-Y	−0.025	0.002	0.012	−0.054	−0.007
Direct Effect	0.084	0.087	0.049	−0.015	0.178
Indirect Effect	−0.052	0.081	0.034	−0.127	0.007
Total Effect	0.032	0.544	0.050	−0.081	0.119
X3-M1-Y	−0.036	0.039	0.019	−0.076	−0.002
X3-M2-Y	−0.141	<0.01	0.040	−0.228	−0.071
X3-M3-Y	−0.016	0.067	0.011	−0.043	0.001
Direct Effect	−0.015	0.795	0.060	−0.136	0.101
Indirect Effect	−0.193	<0.01	0.043	−0.279	−0.112
Total Effect	−0.208	0.003	0.066	−0.337	−0.081

X1, institutional responsibility; X2, physicians’ responsibility; X3, governmental/societal responsibility; M1, emotional exhaustion; M2, cynicism; M3, reduced personal accomplishment; Y, turnover intention.

### Hypothesis testing results

The correlation analysis provided preliminary support for Hypotheses 1–3 at the bivariate level. In the structural model, however, the associations varied across psychological contract responsibility domains and burnout dimensions. For Hypothesis 1, significant negative associations were observed for several responsibility–burnout pathways, but the hypothesized negative associations were not uniformly significant across all responsibility domain–burnout dimension combinations. Therefore, Hypothesis 1 received partial support. For Hypothesis 2, emotional exhaustion, cynicism, and reduced personal accomplishment were all significantly positively associated with turnover intention. Therefore, Hypothesis 2 received full support. For Hypothesis 3, institutional and governmental/societal responsibility fulfillment showed significant negative total associations with turnover intention, whereas physicians’ responsibility fulfillment did not show a significant total association. Therefore, Hypothesis 3 received partial support. Finally, the bootstrap analysis showed differentiated patterns of specific indirect associations across the three responsibility domains and burnout dimensions. Therefore, Hypothesis 4 received full support. These structural associations are presented in Figure 2.

**Figure 2:**
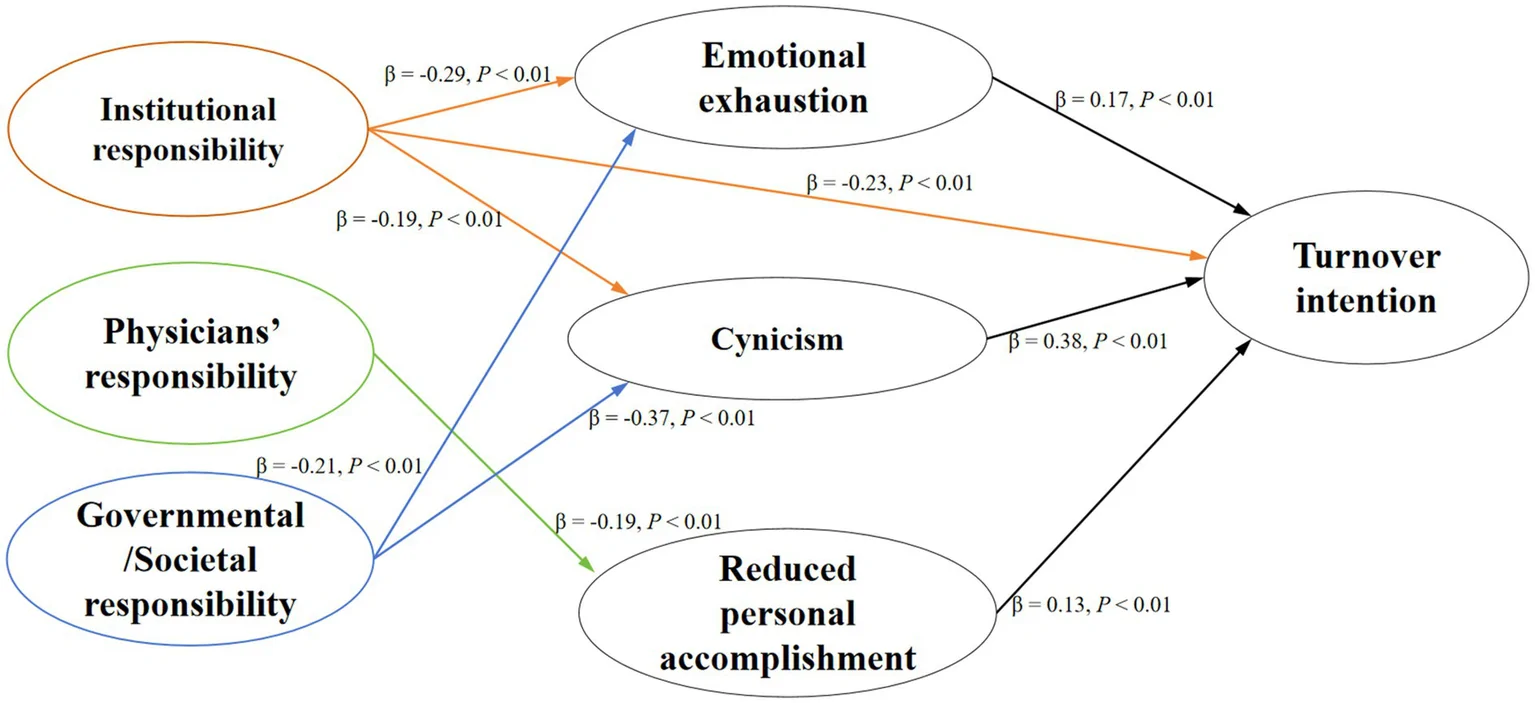
Standardized structural equation model showing associations among psychological contract responsibility fulfillment, burnout dimensions, and turnover intention.

## Discussion

This study examined the structural associations among perceived fulfillment of multilevel psychological contract responsibilities, job burnout dimensions, and turnover intention among family doctors. The findings showed differentiated patterns across the three responsibility domains. Perceived fulfillment of institutional responsibility showed both direct and indirect associations with turnover intention, whereas governmental/societal responsibility was associated with turnover intention primarily through indirect associations involving emotional exhaustion and cynicism. Physicians’ responsibility did not show a significant overall association with turnover intention but exhibited a significant specific indirect association through reduced personal accomplishment. These findings suggest that distinguishing institutional, physicians’, and governmental/societal responsibilities may provide a more differentiated understanding of how psychological contract fulfillment is associated with burnout and turnover-related outcomes in primary care.

Compared with previous structural studies of burnout and turnover intention, the present findings are consistent with evidence that occupational conditions are associated with burnout and subsequent withdrawal-related outcomes (10, 23). However, the present study differs in two respects. First, rather than examining only general job demands, organizational factors, or single-source support, it considers perceived fulfillment of psychological contract responsibilities across three distinct domains: institutional responsibility, physicians’ own responsibility, and governmental/societal responsibility. Second, rather than treating burnout as a single overall construct, emotional exhaustion, cynicism, and reduced personal accomplishment were examined separately. The observed pattern suggests that the three responsibility domains are not uniformly associated with burnout-related processes, thereby extending previous models by combining a multilevel psychological contract perspective with a multidimensional assessment of burnout.

The negative associations between perceived fulfillment of psychological contract responsibilities and burnout are broadly consistent with previous research linking psychological contract perceptions to employees’ emotional and occupational outcomes (33, 55, 57). From a psychological contract perspective, perceived fulfillment indicates that expected reciprocal obligations are being met, whereas insufficient fulfillment may be associated with unfavorable emotional evaluations of the employment relationship. The JD-R model provides a complementary interpretation of these findings: fulfillment of institutional and governmental/societal responsibilities involves work-context conditions such as employment security, working conditions, recognition, professional protection, and policy support, which may have resource-related implications for physicians coping with occupational demands. Importantly, this interpretation does not imply that psychological contract fulfillment itself is equivalent to a job resource.

Our findings confirm that job burnout is a critical correlate of turnover intention among family doctors (58). Notably, cynicism showed a stronger association with the intention to leave than emotional exhaustion. One possible explanation is that the relationship-intensive nature of family medicine may make cynicism particularly relevant to turnover intention. Cynicism involves psychological distancing from work, and such detachment may be especially salient in a professional context characterized by continuing patient and community relationships. However, this interpretation remains hypothetical because relational demands, physician–patient interaction quality, and related interpersonal mechanisms were not directly measured in the present study.

The structural results further showed different patterns for institutional and governmental/societal responsibility fulfillment. Institutional responsibility showed both a significant direct association with turnover intention and specific indirect associations through emotional exhaustion and cynicism. This pattern is broadly consistent with evidence that organizational conditions, perceived support, fairness, and psychological contract-related experiences are associated with withdrawal-related attitudes (59–61). In contrast, governmental/societal responsibility showed no significant direct association with turnover intention, while its total indirect association was significant, primarily through cynicism and emotional exhaustion. This finding suggests that perceptions of broader governmental and societal obligations may be associated with turnover intention more strongly through physicians’ burnout-related experiences than through a direct structural association. Physicians’ responsibility showed a different pattern. Although its total, direct, and total indirect associations with turnover intention were not statistically significant, a significant specific indirect association was observed through reduced personal accomplishment. Because this responsibility domain concerns physicians’ perceptions of the extent to which they themselves fulfill professional obligations, the finding should not be interpreted as evidence of external support or as showing that physicians’ responsibility itself constitutes a personal resource. One possible interpretation is that fulfillment of physicians’ own professional obligations may be related to experiences of competence, value congruence, professional efficacy, or accomplishment, which are conceptually relevant to the reduced-personal-accomplishment dimension of burnout (42, 61). However, professional identity, competence, value congruence, and related personal-resource processes were not directly measured in this study; therefore, this explanation remains hypothetical and requires direct examination in future research. Taken together, these differentiated associations suggest that the three psychological contract responsibility domains should not be treated as interchangeable forms of support. Rather, they represent distinct domains of perceived responsibility fulfillment that may relate differently to specific burnout experiences and turnover intention. The observed pattern is compatible with using the JD-R framework as a complementary explanatory perspective, while retaining psychological contract fulfillment as the construct directly assessed in the study. The cross-sectional nature of the study precludes definitive causal inference and does not establish temporal precedence among psychological contract responsibility fulfillment, burnout, and turnover intention. Reverse or reciprocal associations therefore remain possible. For example, physicians experiencing higher levels of burnout may evaluate the fulfillment of institutional or governmental/societal responsibilities more negatively, while physicians with stronger turnover intentions may retrospectively perceive obligations within their employment relationships as less fulfilled. Perceptions of physicians’ own responsibility fulfillment may likewise vary with their current psychological state and professional evaluations. Accordingly, the observed structural associations should not be interpreted as demonstrating a unidirectional causal process. Longitudinal and cross-lagged studies are needed to clarify the temporal ordering and directionality of these relationships.

### Recommendations

Based on the associations identified in this study, the three psychological contract responsibility domains may provide useful areas for considering potential organizational, policy, and professional implications. Because the present study was cross-sectional, the following recommendations should be interpreted as hypothesis-generating implications rather than as interventions demonstrated to reduce burnout or turnover intention.

Governmental/Societal Level: The findings suggest that perceived fulfillment of governmental/societal responsibilities may be relevant to physicians’ burnout-related experiences, particularly emotional exhaustion and cynicism. Accordingly, health authorities may consider examining policy conditions related to professional protection, social recognition, occupational autonomy, and the broader practice environment of family doctors. However, the present findings do not establish that changes in these policy conditions will reduce burnout or turnover intention; prospective and intervention studies are needed to evaluate such possibilities.

Institutional Level: Perceived fulfillment of institutional responsibility showed both direct and indirect associations with turnover intention, with specific indirect associations involving emotional exhaustion and cynicism. These findings suggest that managers may consider reviewing organizational practices related to transparent resource allocation, career development, working conditions, recognition, and other institutional obligations relevant to family doctors (62–64). Workflow redesign, psychological support, or screening programs may also warrant evaluation as potential approaches to addressing emotional exhaustion; however, their effectiveness was not tested in the present study and should be examined in future intervention research (65).

Physician-Level Implications: Physicians’ responsibility did not show a significant overall association with turnover intention, but a significant specific indirect association was observed through reduced personal accomplishment. Because this responsibility domain reflects physicians’ perceived fulfillment of their own professional obligations, the finding should not be interpreted as evidence that individual psychological resources were directly measured. Nevertheless, professional efficacy, competence, value congruence, and professional identity may represent relevant factors for future investigation. Approaches such as peer supervision, reflective practice, professional development, or interventions aimed at strengthening professional meaning may therefore warrant evaluation, but their effectiveness cannot be inferred from the present findings (66, 67).

### Limitations and future research

This study possesses several notable limitations. First, the cross-sectional and self-reported design limits causal interpretation and may introduce common method bias. Although Harman’s single-factor test did not indicate a dominant single-factor structure, this approach provides only a preliminary assessment of common method bias, and residual bias cannot be completely excluded because all variables were measured using self-reported data from the same respondents. The reliance on self-reported perceptual measures may also have introduced social desirability bias, as participants may have adjusted their responses according to perceived expectations or concerns about their professional image. Furthermore, although bootstrap analysis was used to examine indirect associations among perceived fulfillment of psychological contract responsibilities, burnout dimensions, and turnover intention, the cross-sectional design does not establish temporal precedence among these variables. Therefore, the observed indirect associations should be interpreted as hypothetical statistical associations rather than confirmed causal mediation processes. Future studies using longitudinal designs, multi-source data, or objective organizational indicators are needed to further examine these relationships and reduce potential bias associated with self-reported measures. Second, the sample included a relatively high proportion of female participants (70.0%). Previous studies have similarly reported a relatively high proportion of female physicians among family doctors/primary care physicians (68, 69), suggesting that the female predominance observed in our sample may partly reflect the demographic characteristics of this professional population. However, these studies do not establish the actual gender distribution of primary care physicians in Shandong Province. Therefore, whether the gender composition of our sample fully represents the target population remains uncertain, and potential gender-related selection bias cannot be completely excluded. This limitation may also affect the generalizability of the present findings. Future studies should recruit larger and more diverse samples to further validate these findings. Third, although several demographic and occupational characteristics, such as age, working hours, employment status, salary, incentive policies, and health status, may be associated with burnout and turnover intention (70), these variables were not included as covariates in the structural equation model. Therefore, residual confounding cannot be completely excluded. Future studies should incorporate relevant individual and occupational characteristics into structural models to further examine these relationships. Fourth, although the revised measurement model demonstrated acceptable reliability and validity after item refinement, three items were removed during measurement model refinement because their standardized factor loadings did not meet the recommended threshold. This modification may have affected the original content coverage of the constructs. Future studies should further validate the adapted instrument in diverse primary healthcare settings. Finally, this study was conducted only in Shandong Province, and although multi-stage random sampling was used to include physicians from different administrative levels and primary healthcare institutions, the findings may not be directly generalizable to all regions of China with different healthcare resources, economic conditions, and policy environments. Future studies involving multiple provinces and diverse geographic regions are needed to further evaluate the applicability and generalizability of this framework.

## Conclusion

This study examined the associations among perceived fulfillment of multilevel psychological contract responsibilities, job burnout dimensions, and turnover intention among primary care physicians. Perceived fulfillment of institutional responsibility showed significant direct and indirect associations with turnover intention, whereas governmental/societal responsibility was associated with turnover intention primarily through indirect associations involving emotional exhaustion and cynicism. Physicians’ responsibility did not show a significant overall association with turnover intention but demonstrated a significant specific indirect association through reduced personal accomplishment.

These findings suggest that the three psychological contract responsibility domains are associated with burnout and turnover intention in differentiated ways. Psychological contract theory provides the conceptual basis for defining the responsibility domains, while the JD-R model offers a complementary perspective for interpreting their potential work- and resource-related implications. Given the cross-sectional design, the observed relationships should be interpreted as structural associations rather than causal mechanisms. Longitudinal and intervention studies are needed to determine whether changes in responsibility fulfillment are followed by changes in burnout or turnover intention.

## Data Availability

The raw data supporting the conclusions of this article will be made available by the authors, without undue reservation.

## References

[1] Cai C Hone T Millett C. The heterogeneous effects of China's hierarchical medical system reforms on health service utilisation and health outcomes among elderly populations: a longitudinal quasi-experimental study. Lancet. (2023) 402:S30. doi: 10.1016/S0140-6736(23)02141-4, 37997071

[2] Jiang R Xin Y Peng S Zhou Y Zhang X Shi Y . Facilitators and barriers to chronic non-communicable disease management under family doctor contracting services in China. Front Med. (2025) 12:1506016. doi: 10.3389/fmed.2025.1506016, 40134919 PMC11932984

[3] Flegg KM Martinez-Bianchi V. Caring for the caregivers: a global imperative for family doctors’ wellbeing. Lancet Primary Care. (2025) 1:100030. doi: 10.1016/j.lanprc.2025.100030

[4] Li X Wang J He L Hu Y Li C Xie Y . Turnover intention and influential factors among primary healthcare workers in Guangdong province, China: a cross-sectional study. BMJ Open. (2024) 14:e084859. doi: 10.1136/bmjopen-2024-084859, 39551594 PMC11574511

[5] He R Liu J Zhang W-H Zhu B Zhang N Mao Y. Turnover intention among primary health workers in China: a systematic review and meta-analysis. BMJ Open. (2020) 10:e037117. doi: 10.1136/bmjopen-2020-037117, 33067275 PMC7569945

[6] Shi J Zhou P Xue D Zhang Y Bai F Zhu H . Development of psychological contract scale for physicians in public hospitals. Chin Hosp Manag. (2016) 36:19–21.

[7] Freudenberger HJ. Staff burn-out. J Soc Issues. (1974) 30:159–65. doi: 10.1111/j.1540-4560.1974.tb00706.x

[8] Maslach C Jackson SE. The measurement of experienced burnout. J Organ Behav. (1981) 2:99–113. doi: 10.1002/job.4030020205

[9] Zhang T Feng J Jiang H Shen X Pu B Gan Y. Association of professional identity, job satisfaction and burnout with turnover intention among general practitioners in China: evidence from a national survey. BMC Health Serv Res. (2021) 21:382. doi: 10.1186/S12913-021-06322-6, 33902579 PMC8074426

[10] Ran L Chen X Peng S Zheng F Tan X Duan R. Job burnout and turnover intention among Chinese primary healthcare staff: the mediating effect of satisfaction. BMJ Open. (2020) 10:e036702. doi: 10.1136/bmjopen-2019-036702, 33033013 PMC7542935

[11] Tawfik DS Scheid A Profit J Shanafelt T Trockel M Adair KC . Evidence relating health care provider burnout and quality of care: a systematic review and meta-analysis. Ann Intern Med. (2019) 171:555–67. doi: 10.7326/M19-1152, 31590181 PMC7138707

[12] Li LZ Yang P Singer SJ Pfeffer J Mathur MB Shanafelt T. Nurse burnout and patient safety, satisfaction, and quality of care: a systematic review and meta-analysis. JAMA Netw Open. (2024) 7:e2443059. doi: 10.1001/JAMANETWORKOPEN.2024.43059, 39499515 PMC11539016

[13] Wen Z Qin Y Ni Y Wang Y Xu K Zhu Y. Burnout and turnover intention in primary public health workers: the mediating role of job satisfaction. Front Public Health. (2025) 13:1708432. doi: 10.3389/FPUBH.2025.1708432, 41626367 PMC12852422

[14] Rousseau D. Psychological Contracts in Organizations: Understanding Written and Unwritten Agreements. Thousand Oaks, California: SAGE Publications, Inc.; (1995). doi: 10.4135/9781452231594

[15] Zhang X Chen X Chen C Wang Y Shindo K Zhang X. The influence mechanism of psychological contract on primary medical staff's turnover intention in the context of COVID-19 pandemic in China. Int J Health Plann Manag. (2022) 37:2936–48. doi: 10.1002/hpm.3533, 35709222 PMC9349371

[16] Laulié L Briceño-Jiménez G Henríquez-Gómez G. Exploring self-regulation theory as a mechanism of the effects of psychological contract fulfillment: the role of emotional intelligence. Front Psychol. (2023) 14:1090094. doi: 10.3389/FPSYG.2023.1090094, 37063526 PMC10098163

[17] Sischka PE Melzer A Schmidt AF Steffgen G. Psychological contract violation or basic need frustration? Psychological mechanisms behind the effects of workplace bullying. Front Psychol. (2021) 12:627968. doi: 10.3389/FPSYG.2021.627968, 33897535 PMC8062865

[18] Xanthopoulou D Bakker AB Demerouti E Schaufeli WB. The role of personal resources in the job demands-resources model. Int J Stress Manag. (2007) 14:121–41. doi: 10.1037/1072-5245.14.2.121

[19] Lesener T Gusy B Wolter C. The job demands-resources model: a meta-analytic review of longitudinal studies. Work Stress. (2019) 33:76–103. doi: 10.1080/02678373.2018.1529065

[20] Bakker AB Demerouti E. The job demands-resources model: state of the art. J Manager Psychol. (2007) 22:309–28. doi: 10.1108/02683940710733115

[21] Eisenberger R Huntington R Hutchison S Sowa D. Perceived Organizational Support. *J Appl Psychol.* (1986). 71:500–7. doi: 10.1037/0021-9010.71.3.500

[22] Wang T Abrantes ACM Liu Y. Intensive care units nurses' burnout, organizational commitment, turnover intention and hospital workplace violence: a cross-sectional study. Nurs Open. (2023) 10:1102–15. doi: 10.1002/NOP2.1378, 36126210 PMC9834521

[23] Park SK Rhee M-K Lee SW. The effects of job demands and resources on turnover intention: the mediating roles of emotional exhaustion and depersonalization. Work. (2021) 70:301–9. doi: 10.3233/WOR-213574, 34511473

[24] Shen X Jiang H Xu H Ye J Lv C Lu Z . The global prevalence of turnover intention among general practitioners: a systematic review and meta-analysis. BMC Fam Pract. (2020) 21:246. doi: 10.1186/s12875-020-01309-4, 33250045 PMC7702723

[25] Sun Y Wang W Yan F Xie X Cai M Xu F . Related factors of turnover intention among general practitioners: a cross-sectional study in 6 provinces of China. BMC Prim Care. (2025) 26:37. doi: 10.1186/S12875-025-02728-X, 39939970 PMC11816535

[26] Mobley WH Horner SO Hollingsworth AT. An evaluation of precursors of hospital employee turnover. J Appl Psychol. (1978) 63:408–14. doi: 10.1037/0021-9010.63.4.408 701211

[27] Hom PW Lee TW Shaw JD Hausknecht JP. One hundred years of employee turnover theory and research. J Appl Psychol. (2017) 102:530–45. doi: 10.1037/apl0000103 28125259

[28] Kim H Kim EG. A meta-analysis on predictors of turnover intention of hospital nurses in South Korea (2000–2020). Nurs Open. (2000) 8:2406–18. doi: 10.1002/nop2.872, 33826252 PMC8363357

[29] Hur Y. Assessing the effects of workplace contextual factors on turnover intention: evidence from US federal employees. Publ Organ Rev. (2025) 25:193–213. doi: 10.1007/S11115-024-00784-Y

[30] Wen T Zhang Y Wang X Tang G. Factors influencing turnover intention among primary care doctors: a cross-sectional study in Chongqing, China. Hum Resour Health. (2018) 16:10. doi: 10.1186/s12960-018-0274-z, 29433519 PMC5809822

[31] Lu Y Hu X-M Huang X-L Zhuang X-D Guo P Feng L-F . The relationship between job satisfaction, work stress, work–family conflict, and turnover intention among physicians in Guangdong, China: a cross-sectional study. BMJ Open. (2017) 7:e014894. doi: 10.1136/bmjopen-2016-014894, 28501813 PMC5566636

[32] Topa G Aranda-Carmena M De-Maria B. Psychological contract breach and outcomes: a systematic review of reviews. Int J Environ Res Public Health. (2022) 19:15527. doi: 10.3390/IJERPH192315527, 36497602 PMC9737235

[33] Qadri SU Li M Ma Z Qadri S Ye C Usman M. Unpaid leave on COVID-19: the impact of psychological breach contract on emotional exhaustion: the mediating role of job distrust and insecurity. Front Psychol. (2022) 13:953454. doi: 10.3389/FPSYG.2022.953454, 35936340 PMC9355467

[34] He Z Chen L Shafait Z. How psychological contract violation impacts turnover intentions of knowledge workers? The moderating effect of job embeddedness. Heliyon. (2023) 9:e14409. doi: 10.1016/j.heliyon.2023.e14409, 36950653 PMC10025906

[35] Bal PM De Cooman R Mol ST. Dynamics of psychological contracts with work engagement and turnover intention: the influence of organizational tenure. Eur J Work Organ Psychol. (2013) 22:107–22. doi: 10.1080/1359432X.2011.626198

[36] Lu X Yang J Bai D Cai M Wang W He J . The effect of psychological contract on turnover intention among nurses: a meta-analytic review. BMC Nurs. (2023) 22:358. doi: 10.1186/s12912-023-01496-2, 37798645 PMC10557294

[37] Van der Heijden B Brown Mahoney C Xu Y. Impact of job demands and resources on nurses’ burnout and occupational turnover intention towards an age-moderated mediation model for the nursing profession. Int J Environ Res Public Health. (2019) 16:2011. doi: 10.3390/ijerph16112011, 31195753 PMC6604012

[38] Maslach C Schaufeli WB Leiter MP. Job burnout. Annu Rev Psychol. (2001) 52:397–422. doi: 10.1146/annurev.psych.52.1.397, 11148311

[39] Gluschkoff K Hakanen J Elovainio M Vänskä J Heponiemi T. The relative importance of work-related psychosocial factors in physician burnout. Occup Med (Lond). (2022) 72:28–33. doi: 10.1093/occmed/kqab147, 34729593 PMC8758190

[40] Lee RT Seo B Hladkyj S Lovell BL Schwartzmann L. Correlates of physician burnout across regions and specialties: a meta-analysis. Hum Resour Health. (2013) 11:48. doi: 10.1186/1478-4491-11-48, 24074053 PMC3849515

[41] Leiter MP Frank E Matheson TJ. Demands, values, and burnout: relevance for physicians. Can Fam Physician. (2009) 55:1224–5. e6. PMC2793232 20008605

[42] Yoshia M Ken-Ei S Kenta S Shoichi N Yosuke A Yu K . The association of grit with burnout components (professional efficacy, exhaustion, and cynicism) among academic rheumatologists. J Clin Rheumatol. (2023) 29:268–74. doi: 10.1097/RHU.0000000000001989 37226300

[43] Shandong Provincial Bureau of Statistics, National Bureau of Statistics Shandong Survey Office. 2025 Shandong Province national economic and social Development Statistics Bulletin. Available online at: http://tjj.shandong.gov.cn/art/2026/3/3/art_6196_10322526.html (Accessed March 3, 2026).

[44] Li C Shi K. The influence of distributive justice and procedural justice on job burnout. Acta Psychol Sin. (2003) 5:677–84.

[45] Li H. The Relationship between Psychological Resilience and job Burnout among Psychiatric Nurses [Master's thesis]. Jinan, China: Shandong University (2014).

[46] Ren Y Li G Pu D He L Huang X Lu Q . The relationship between perceived organizational support and burnout in newly graduated nurses from Southwest China: the chain mediating roles of psychological capital and work engagement. BMC Nurs. (2024) 23:719. doi: 10.1186/s12912-024-02386-x, 39379880 PMC11459715

[47] Farh J-L Tsui AS Xin K Cheng B-S. The influence of relational demography and guanxi: the Chinese case. Organ Sci. (1998) 9:471–88. doi: 10.1287/orsc.9.4.471

[48] Zeng X Liu C. An empirical investigation and reflection on turnover intention of young university teachers. J High Educ Manag. (2015) 9:100–5.

[49] Podsakoff PM MacKenzie SB Lee J-Y Podsakoff NP. Common method biases in behavioral research: a critical review of the literature and recommended remedies. J Appl Psychol. (2003) 88:879–903. doi: 10.1037/0021-9010.88.5.879, 14516251

[50] Taber KS. The use of Cronbach’s alpha when developing and reporting research instruments in science education. Res Sci Educ. (2018) 48:1273–96. doi: 10.1007/s11165-016-9602-2

[51] Fornell C Larcker DF. Evaluating structural equation models with unobservable variables and measurement error. J Mark Res. (1981) 18:39–50. doi: 10.1177/002224378101800104

[52] Cheung GW Cooper-Thomas HD Lau RS Wang LC. Reporting reliability, convergent and discriminant validity with structural equation modeling: a review and best-practice recommendations. Asia Pac J Manag. (2024) 41:745–83. doi: 10.1007/s10490-023-09871-y

[53] Kline RB. Principles and Practice of Structural Equation Modeling. New York, NY: Guilford Publications (2023).

[54] Bakker AB De Vries JD. Job demands–resources theory and self-regulation: new explanations and remedies for job burnout. Anxiety Stress Coping. (2021) 34:1–21. doi: 10.1080/10615806.2020.1797695, 32856957

[55] Yu D Yang K Zhao X Liu Y Wang S D’Agostino MT . Psychological contract breach and job performance of new generation of employees: considering the mediating effect of job burnout and the moderating effect of past breach experience. Front Psychol. (2022) 13:985604. doi: 10.3389/FPSYG.2022.985604, 36160600 PMC9501888

[56] Cai X Zhao T Chen L Zhang S Yu A Sun X . Job burnout among primary healthcare workers during COVID-19 pandemic: cross-sectional study in China. Front Public Health. (2023) 11:1266864. doi: 10.3389/FPUBH.2023.1266864, 38125852 PMC10731250

[57] Delegach M Klein G Katz-Navon T. Furlough and its effects on employees after returning to work: the roles of psychological contract breach and violation, and perceived organizational support. J Manag Organ. (2024) 30:1408–25. doi: 10.1017/JMO.2022.71

[58] Rotenstein LS Torre M Ramos MA Rosales RC Guille C Sen S . Prevalence of burnout among physicians: a systematic review. JAMA. (2018) 320:1131–50. doi: 10.1001/jama.2018.12777, 30326495 PMC6233645

[59] Park J Wolfart R King J Sicam A Viswesvaran C. You betrayed me: the role of support in the psychological contract breach and turnover intention link. J Manag Organ. (2025) 31:2511–26. doi: 10.1017/JMO.2025.10013

[60] Zhao H Chen Y Xu Y Zhou Q. Socially responsible human resource management and employees' turnover intention: the effect of psychological contract violation and moral identity. J Manag Organ. (2022) 28:1071–90. doi: 10.1017/JMO.2022.16

[61] Wang Q Wang C. Reducing turnover intention: perceived organizational support for frontline employees. Front Bus Res China. (2020) 14:6. doi: 10.1186/s11782-020-00074-6

[62] Cropanzano R Anthony E Daniels S Hall A. Social exchange theory: a critical review with theoretical remedies. Acad Manag Ann. (2015) 11:479–516. doi: 10.5465/annals.2015.0099

[63] Solinger ON Hofmans J Bal PM Jansen PG. Bouncing back from psychological contract breach: how commitment recovers over time. J Organ Behav. (2016) 37:494–514. doi: 10.1002/job.2047

[64] Yu J. Impacts of psychological contract fulfillment on work attitudes and behaviors during the COVID-19 pandemic: mediating role of perceived organizational support. Curr Psychol. (2024) 43:14851–60. doi: 10.1007/S12144-022-03746-Z, 36340890 PMC9628416

[65] Rehder K Adair KC Sexton JB. The science of health care worker burnout: assessing and improving health care worker well-being. Arch Pathol Lab Med. (2021) 145:1095–109. doi: 10.5858/arpa.2020-0557-RA, 34459858

[66] Zhang X Chen S Zheng Z Zhao M Song L Zhao Y . The relationship between psychological capital, burnout and perceived stress in junior nurses: a latent profile analysis. Front Public Health. (2024) 12:1374941. doi: 10.3389/fpubh.2024.1374941 PMC11039913 38660345

[67] Hiefner AR Constable P Ross K Sepdham D Ventimiglia JB. Protecting family physicians from burnout: meaningful patient-physician relationships are “more than just medicine”. J Am Board Fam Med. (2022) 35:716–23. doi: 10.3122/JABFM.2022.04.210441, 35896474

[68] Feng H Jing R Wang J Fang H. Research on incentive factors of family doctor team members in different positions. Chin Gen Pract. (2021) 24:400–6. doi: 10.12114/j.issn.1007-9572.2021.00.089

[69] Huang C Lan C Chen Z Yu Y Gao J Yang H. Knowledge, attitude and practice regarding screening and managing diabetic microvascular complications among general practitioners of community health centres: a cross-sectional study in Shanghai, China. BMJ Open. (2025) 15:e085784. doi: 10.1136/bmjopen-2024-085784, 39890145 PMC11784367

[70] West CP Dyrbye LN Shanafelt TD. Physician burnout: contributors, consequences and solutions. J Intern Med. (2018) 283:516–29. doi: 10.1111/joim.12752, 29505159

